# Dihydroartemisinin inhibits TCTP-dependent metastasis in gallbladder cancer

**DOI:** 10.1186/s13046-017-0531-3

**Published:** 2017-05-15

**Authors:** Fei Zhang, Qiang Ma, Zihang Xu, Haibin Liang, Huaifeng Li, Yuanyuan Ye, Shanshan Xiang, Yijian Zhang, Lin Jiang, Yunping Hu, Zheng Wang, Xuefeng Wang, Yong Zhang, Wei Gong, Yingbin Liu

**Affiliations:** 1 Department of General Surgery, Xinhua Hospital affiliated to Shanghai Jiao Tong University School of Medicine, Room 517, Building 22, Xinhua Hospital, 1665 Kongjiang Rd., Shanghai, 200092 China; 2 Shanghai Research Center of Biliary Tract Disease, 1665 Kongjiang Road, Shanghai, 200092 China; 3 Laboratory of Integrative Medicine, School of Basic Medical Sciences, Shanghai University of Traditional Chinese Medicine, 1200 Cailun Road, Shanghai, 201203 China

**Keywords:** Metastasis, Invasion, Gallbladder cancer, TCTP, Dihydroartemisinin

## Abstract

**Background:**

Patients with metastatic or relapsed gallbladder cancer generally have a poor prognosis. Therefore, targeting metastasis is one arm of therapeutic strategies to treat gallbladder cancer.

**Methods:**

Levels of translationally controlled tumor protein (TCTP) were measured in samples of gallbladder cancer by immunohistochemical staining. Wound healing, migration and invasion assays were used to investigate the motility of cells. Western blot assay was used to investigate the levels of TCTP and other proteins. Liver metastasis models and lung metastasis models were established to investigate the inhibitory effect of Dihydroartemisinin on gallbladder cancer metastasis.

**Results:**

TCTP is aberrantly expressed in gallbladder cancer patients and associated with metastasis and a poor prognosis. Depleting TCTP significantly inhibited gallbladder cancer cell migration and invasion. We found that Dihydroartemisinin as a potent inhibitor of TCTP inhibited TCTP-dependent cell migration and invasion by reducing cell division control protein 42 homolog (Cdc42) activation. In addition, in mice with xenografted tumors, treatment with Dihydroartemisinin decreased gallbladder cancer cell metastases and improved survival.

**Conclusions:**

These findings provide new insights into the therapeutic activity of Dihydroartemisinin as a treatment for gallbladder cancer metastasis.

**Electronic supplementary material:**

The online version of this article (doi:10.1186/s13046-017-0531-3) contains supplementary material, which is available to authorized users.

## Background

Gallbladder cancer (GBC) is the most malignant type of biliary tract cancer. It is also the seventh most common gastrointestinal cancer, with an incidence of 2.5 per 100,000 persons [1–3]. The poor prognosis associated with this deadly disease results in large part from its late detection, its rapidly metastatic invasiveness, and its ability to acquire resistance to conventional chemotherapies [4, 5]. Average survival is less than 1 year, even in patients treated with adjuvant therapy with standard chemotherapeutic agents [6, 7]. Therefore, new therapeutic approaches that target the key proteins that mediate metastasis are urgently needed to treat this aggressive tumor.

It is well known that metastasis is a major cause of cancer lethality, accounting for as many as 90% of all cancer deaths. Strikingly, recent data demonstrate that metastasis occurs early in the development of GBC, even prior to the formation of the primary tumor [8]. While one goal of cytotoxic therapies is to minimize metastatic tumor growth, therapies specifically designed to inhibit tumor migration and invasion, which underly metastasis, are not currently available [9]. Therefore, blocking metastasis should be considered one arm of therapeutic strategies aimed at treating GBC, even in the absence of detectable metastatic lesions.

Translationally controlled tumor protein (TCTP) is a highly conserved protein that plays important roles in numerous cellular physiological events, such as immune responses, gene regulation, cell proliferation, apoptosis, and tumorigenesis [10]. In cancer, TCTP is known to regulate tumor suppressor gene p53 [11] and activate components of the mTOR pathway [12] during both the subversion of the cancer stem cell compartment and the tumor reversion program [13]. Recently, a growing amount of clinical evidence indicates that TCTP is over-expressed in multiple cancers and associated with cancer metastasis [11, 14]. Lee et al. reported that TCTP triggered tumor cells, causing them to undergo epithelial to mesenchymal transition, and promoted cell migration, invasion and metastasis [15]. TCTP was mediated with colorectal cancer progression and liver metastasis [16]. Our previous study also showed that a high level of TCTP expression significantly correlated with nodal metastasis in pancreatic cancer [17]. In this study, we found that the TCTP protein was aberrantly expressed in GBC and that its expression was correlated with metastasis and poor prognoses in GBC patients. Moreover, depleting TCTP significantly inhibited GBC cell migration and invasion. These results may indicate that TCTP is a potent therapeutic target for inhibiting metastasis in GBC cancer.

Dihydroartemisinin (DHA) is a metabolite of Artemisinin, which is the active ingredient in *Artemisia annua*. DHA is a well-established, well-tolerated drug that is used as an anti-malarial agent and has also been demonstrated to possess anticancer activity [18, 19]. Although DHA has been used in a clinical setting for decades, it has recently been demonstrated that DHA can bind and potently inhibit human TCTP [20, 21]. Because DHA has an inhibitory effect on TCTP function, we hypothesized that DHA could be used to reduce TCTP’s functions in the migration and invasion of GBC cells. The objective of this study was to determine whether DHA could be used as an anti-metastatic agent in GBC patients with TCTP-positive tumors. Here, we show that using DHA to inhibit TCTP-mediated migration significantly disrupted the metastatic potential of GBC cells. These findings reveal the novel therapeutic value of using DHA, a classic anti-malaria drug, to treat patients with GBC.

## Methods

### Cell culture and reagents

The human GBC cell lines NOZ, EH-GB-1, EH-GB-2, SGC-996 and GBC-SD were purchased from the Cell Bank of Type Culture Collection of the Chinese Academy of Sciences (Shanghai, China). The OCUG-1 cell line was obtained from the Health Science Research Resources Bank (Osaka, Japan). The NOZ cell line was maintained in William’s medium (Gibco, Grand Island, NY, USA) supplemented with 10% fetal bovine serum (FBS; Gibco). The GBC-SD cells were maintained in DMEM medium (Gibco). The EH-GB-1, EH-GB-2, SGC-996 and OCUG-1 cells were cultured in RPMI 1640 (Gibco). DHA was purchased from Selleck Chemicals and dissolved in DMSO. Primary antibodies against TCTP, vinculin, paxillin and β-actin were purchased from Cell Signaling Technology (Beverly, MA, USA).

### Patients and clinicopathological data

Tumor tissue specimens were obtained from 73 GBC patients without any prior radiotherapy or chemotherapy who had undergone radical cholecystectomy between 2008 and 2014 at the Department of General Surgery, Xinhua Hospital, School of Medicine, Shanghai Jiaotong University, China. Additionally, 103 patients with cholelithiasis who underwent simple cholecystectomy were included as controls. The patients provided consent for their tumor tissues to be used for clinical research, and the Shanghai Jiaotong University Xinhua Hospital Ethical Committee approved the research protocol. All diagnoses of GBC, cholelithiasis, and lymph node metastasis were confirmed via histopathological examination, and all tissue samples were fixed in 4% formalin immediately after removal and then embedded in paraffin for immunohistochemical staining.

### Immunohistochemical analysis of GBC tissues

After the tissues were sections, deparaffinized and quenched to block endogenous peroxidase activity, they were incubated in 1% bovine serum albumin (BSA) in PBS. The slides were subsequently incubated first with rabbit anti-human-TCTP antibodies (1:500, CST) and then with goat anti-rabbit IgG antibodies. The slides were counterstained with ChemMate Hematoxylin (DakoCytomation) and mounted and observed under a microscope (Olympus). The TCTP expression via IHC was blinded read by two pathologists independently. The sections were semi-quantitatively scored according to the amount and level of immunoreactivity, as follows: 0 (less than 5% positive cells), 1 (5–25% positive cells), 2 (25–50% positive cells), and 3 (greater than 50% positive cells); and 0 (no coloration); 1 (pale yellow); 2 (yellow); and 3 (reddish-brown). The two scores were then multiplied to achieve the following composite scores: 0, negative (−); 1–3, weak (+); 4–6, moderate (++); and 7–9, strong (+++).

### Real-time quantitative PCR (qRT-PCR)

Total RNA was extracted using Trizol LS reagent (Invitrogen). The mRNA was reverse-transcribed using a SuperScript First-Strand Synthesis System (Invitrogen). PCR amplification was performed using the following primers: TCTP, 5′- TATTGGACTACCGTGAGG -3′ (sense) and 5′- CTCGGTGGAAGGACAAACTC -3′ (antisense); and β-actin, 5′-TTAGTTGCGTTACACCCTTTC-3′ (sense) and 5′-ACCTTCACCGTTCCAGTTT3′ (antisense). Real-time quantitative RT-PCR was performed following a standard SYBR Green PCR protocol using SYBR® Premix Ex Taq™ (Takara, Dalian, China). The relative level of TCTP mRNA was normalized to the housekeeping gene β-actin.

### Wound healing assay

GBC cells were seeded into 6-well plates (Becton Dickinson). After the cells had grown to 100% confluence, a wound was made by scraping a conventional pipette tip across the monolayer. The cells were washed in PBS and placed in medium supplemented with or without 10% FBS. After the cells were incubated for 18 h at 37 °C, they were fixed and photographed.

### Cell migration and invasion assays

Cell transwell migration and invasion assay were performed using Boyden chambers (BD Biosciences). The cells (NOZ, GBC-SD, and OCUG-1 cells at 2 × 10^4^ cells/well and EH-GB-2 and SGC-996 cells at 3 × 10^4^ cells/well) were seeded in the upper chamber in DMEM without FBS. In the lower chamber, 600 μl of DMEM containing 10% FBS was added. After the cells were incubated for 24 h and then fixed and stained with crystal violet. The cells in the upper chamber were removed, and three randomly selected fields of cells migrating through the membrane were photographed and counted using ImageJ software (NIH).

### Transfection for knockdown and overexpression experiments

GBC cells were transfected to create TCTP-overexpressing or -knockdown cells, as previously described [17]. A short interfering RNA sequence that targeted Cdc42 (5′- TTCAGCAATGCAGACAATTAA-3′) was designed and synthesized by GenePharma (Shanghai Co., Ltd.). RNAi targeting human Cdc42 and a non-targeting control sequence were transfected using lip2000 (Invitrogen) according to the manufacturer’s instructions. A plasmid encoding myc-tagged active Cdc42 Q61L was purchased from Addgene (Addgene, Cambridge, USA).

### Western blot analysis

GBC cell lines were treated with 40 μM DHA for 48 h, and whole-cell lysates were prepared for western blot analysis according to a previous report [22].

### Immunofluorescence analysis

GBC cells were seeded onto glass slides and treated with DHA for 48 h. The cells were then washed once with PBS, fixed with 4% paraformaldehyde in PBS for 20 min, permeabilized with 0.1% Triton X-100 in PBS for 30 min at room temperature, and then blocked in 3% BSA in PBS for 1 h. The cells were then stained using an anti-Vinculin antibody (1:100 dilution, overnight at 4 °C). The cells were stained for F-actin using phalloidin (Beyotime Biotechnology). Cell nuclei were stained using DAPI, and the samples were mounted using DAPI Fluoromount-G media (Southern Biotech). The slides were viewed using an Olympus confocal microscope (Leica, Switzerland).

### Cell adhesion and spreading

GBC cells were treated with DHA for 48 h, and 1 × 10^4^ cells were then resuspended in Opti-MEM and seeded on fibronectin-coated (25 mg/mL) coverslips for up to 6 h. The cells were fixed and processed for immunofluorescence staining and then examined using confocal microscopy. For cell spreading, the sizes of at least 50 cells were measured using Volocity software (PerkinElmer). To analyze cell adhesion, the number of cells that were attached to the surface was counted in three randomly selected fields under 40 × magnification.

### In vivo metastasis assay

Female Balb/c SCID mice (6–8 weeks of age, weight 18–20 g) were purchased from the Shanghai Laboratory Animal Center of the Chinese Academy of Sciences (Shanghai, China). All mice were housed in specific pathogen-free (SPF) conditions according to the guidelines of the Ethics Committee of Xinhua Hospital, School of Medicine, Shanghai Jiaotong University. To study the effect of DHA on GBC cell metastasis in vivo, we used liver metastasis and lung metastasis mouse models. To induce liver metastasis, the spleens of the mice were injected with 2 × 10^6^ NOZ or EH-GB-2 cells. DHA was transfused via daily intraperitoneal injections, starting on the day after the surgical operation at a dose of 50 mg/kg. The control group was injected with PBS. After 2 months, the mice were euthanized, and the livers were removed for HE staining. In the lung metastasis model, NOZ and EH-GB-2 cells were infected with a lentiviral luciferase vector to generate NOZ-luciferase and EH-GB-2-luciferase cells. A total of 1 × 10^6^ cells of each of these lines were then injected into the mice via the lateral tail vein. DHA was transfused via daily intraperitoneal injections starting on the day after injection at a dose of 50 mg/kg. The trace of the fluorescent cells was monitored every 2 weeks using an IVIS Lumina II (Caliper Life Sciences, USA). The survival times were recorded for 90 days. The mice were sacrificed by CO_2_ inhalation or pentobarbital injection when they had lost >10% of their body weight or when they became moribund (Fatal Plus). To observe tumor metastasis in lung, mice lungs were injected with 1 ml of 15% India ink (Yasutomo, San Francisco, CA) to visualize individual tumor nodules using a standard protocol.

### Statistical analysis

Mann–Whitney *U* test was used to compare TCTP expression between the GBC patients. Kaplan-Meier plots were used for the survival analysis. All data are expressed as the mean values ± standard errors of at least three independent experiments. Statistical significance was calculated using the Mann–Whitney *U* test, and a p value less than 0.05 was considered significant in all tests. All analyses were performed using SPSS software version 19.0 (SPSS Inc., Chicago, IL, United States).

## Results

### TCTP is associated with gallbladder cancer metastasis

**Fig. 1:**
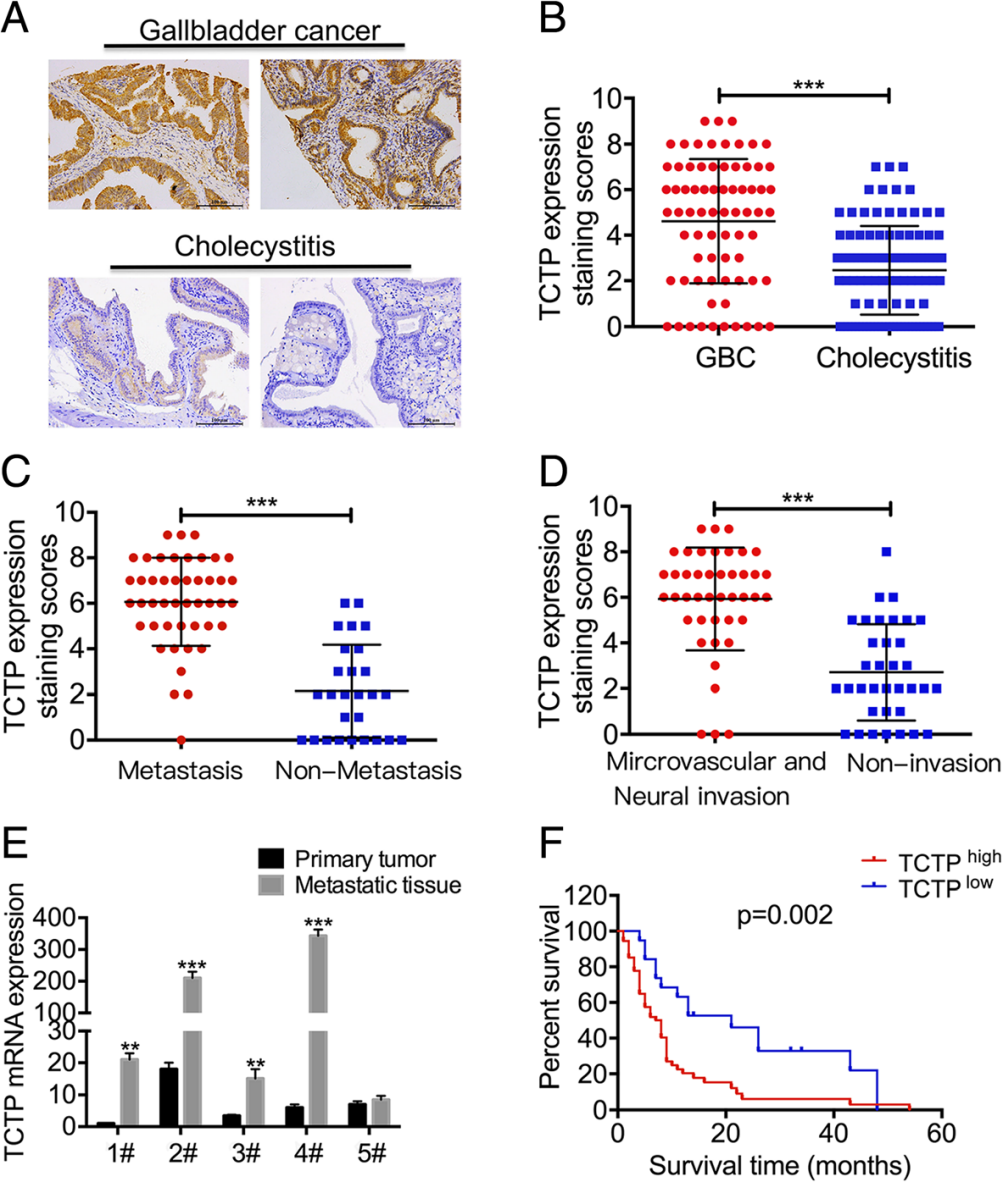
TCTP is associated with gallbladder cancer metastasis. **a** The expression levels of TCTP were detected in 73 gallbladder cancer (GBC) specimens and 103 cholecystitis tissue using IHC staining. Representative IHC images of TCTP expression are shown. **b** The average staining scores for TCTP expression in GBC and cholecystitis tissues were measured using IHC. ***, *p* < 0.001, Mann–Whitney *U* test. **c** TCTP IHC staining scores for non-metastatic and metastatic GBC tissues obtained from patients. ***, *p* < 0.001, Mann–Whitney *U* test. **d** IHC staining scores for TCTP expression in microvascular and neural invasive and non-invasive tissue samples obtained from GBC patients. ***, *p* < 0.001, Mann–Whitney *U* test. **e** TCTP mRNA levels were detected using qPCR in 5 primary tumor and metastatic lymph node samples. **f** Kaplan–Meier plots of the overall survival of GBC patients based on TCTP-high (*n* = 54) or low (*n* = 19) level expression

To determine the role of TCTP in GBC progression, we used IHC to detect TCTP expression levels in 73 GBC specimens and 103 cholecystitis tissues (used as controls). More than 85% of the GBC specimens showed positive expression of TCTP (Additional file 1: Figure S1A). Despite the presence of inter-individual variation, TCTP protein levels were higher in GBC samples than in controls (Fig. 1a and statistical data, Fig. 1b). Furthermore, TCTP was expressed at higher levels in metastatic (including liver, lymph node and abdominal metastases) and invasive (including mircrovascular and neural invasion) GBC samples than in non-metastatic and non-invasive ones (Fig. 1c and d). We were particularly interested in evaluating the difference in TCTP expression levels between primary and metastatic tumors. We therefore obtained primary tumors with metastatic lymph nodes from 5 individual patients and sought to determine their TCTP mRNA expression levels using quantitative RT-PCR. In four out of five of these cases, the mRNA expression level of TCTP was noticeably higher in metastatic lymph nodes than in corresponding primary tumor tissues (Fig. 1e). To determine whether this increase in the expression of TCTP in tumors is potentially associated with reduced patient survival, we separated the GBC patients into the two following groups: 54 cases with high TCTP expression and 19 cases with low TCTP expression. As shown in Fig. 1f, the expression level of TCTP was negatively associated with patient survival. All of these data suggest that an increase in tumor expression of TCTP is associated with metastasis in patients with GBC.

### TCTP promotes GBC cell migration and invasion

**Fig. 2:**
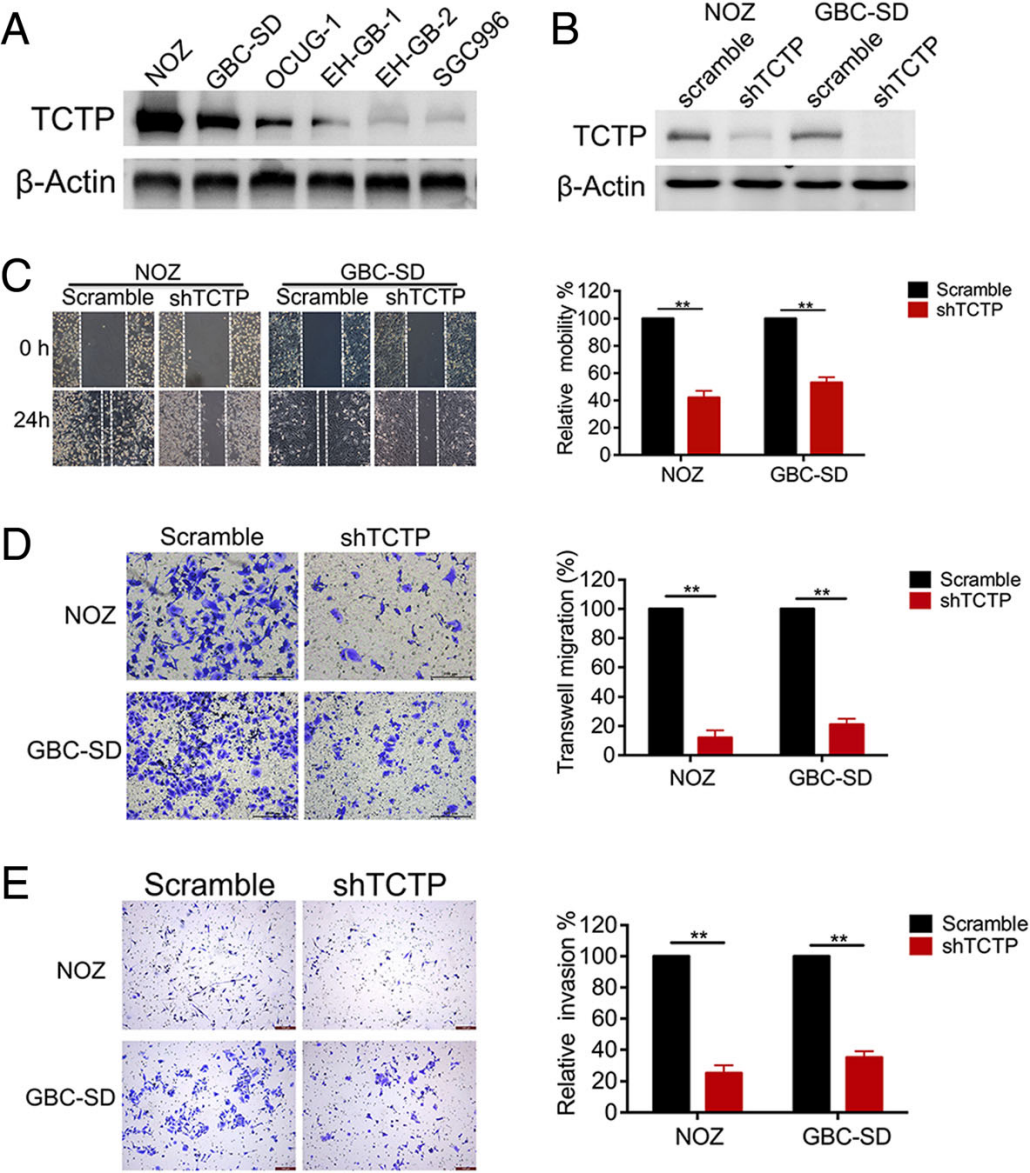
TCTP promotes GBC cell migration and invasion. **a** Western blot analysis of endogenous TCTP expression in six GBC cell lines. β-Actin was used as a loading control. **b** Analysis of TCTP expression in control and TCTP knock-down GBC cells. **c-e** Wound-healing, migration and invasion assays were used to compare GBC cells transfected with either scramble or TCTP shRNA. Cell motility, migration and invasion were quantified by setting the observations in the scramble controls as 100%. Each experiment was independently performed three times, and the graphed data represent the mean ± SD. **, *p* < 0.01

To further investigate the role of TCTP in GBC metastasis, we sought to determine the effect of depleting TCTP on GBC cell migration and invasion. We used shRNA transfection to knock down TCTP expression in the GBC cell lines NOZ and GBC-SD, which express high endogenous levels of TCTP (Fig. 2a). The shRNA efficiently knocked down TCTP expression by more than 90% (Fig. 2b) and was therefore used in subsequent functional studies. In the wound-healing assays, TCTP silencing blocked the migratory activity of NOZ and GBC-SD cells by 40–50% (Fig. 2c). Similarly, depleting TCTP in NOZ and GBC-SD cells resulted in decreases in cell migration and invasion by 80–90% and 65–70%, respectively, in comparison to scramble-treated control cells (Fig. 2d and e). These data suggest that TCTP is critical for migration and invasion in GBC cells.

### DHA inhibits TCTP-dependent cell migration and invasion

**Fig. 3:**
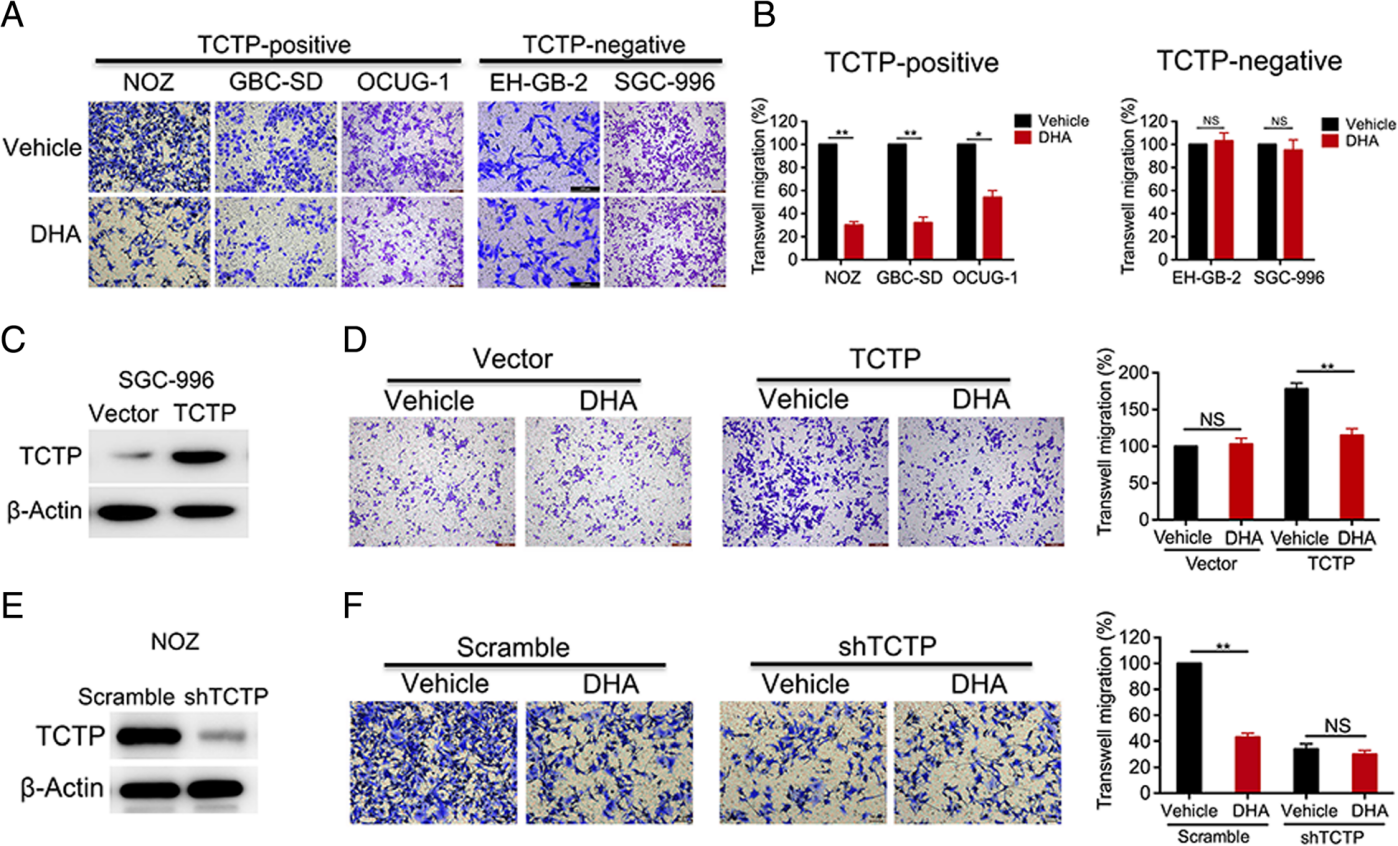
DHA inhibits TCTP-dependent cell migration and invasion. **a** In the migration assays, the TCTP-positive cell lines NOZ, GBC-SD, and OCUG-1, and the TCTP-negative cell lines EH-GB-2 and SGC-996 were pre-treated with either vehicle or DHA (40 μM) for 2 days and then seeded in transwell plates for 24 h. **b** The relative migration rates are shown in a bar graph. **c, d** SGC-996 cells were transfected with an empty or TCTP expression vector (**c**), treated with vehicle or DHA for 2 days, and then seeded in transwell plates for migration assays. The relative migration rates are shown in a bar graph (**d**). **e, f** TCTP was depleted in NOZ cells using shRNA (**e**). The cells were then treated with DHA for 2 days and seeded in transwell plates for migration assays. The relative migration rates are shown in a bar graph (**f**). The percentage of cells that migrated was scored and normalized to the percentage of migrated vehicle-treated cells. The graphed data represent the mean ± SD of 3 independent experiments. *, *p* < 0.05; **, *p* < 0.01, ns: no significant difference (*p* > 0.05)

Based on these findings, we next sought to determine whether inhibiting TCTP would reduce the migratory and invasive potential of GBC. DHA, a drug that has previously been used to treat malaria, has been shown to inhibit TCTP [20] (Additional file 1: Figure S1B, C). To test the hypothesis that DHA inhibits TCTP-mediated cell migration and invasion, we first assessed cell migration in multiple GBC lines, including the TCTP-positive cell lines NOZ, GBC-SD, OCUG-1, and EH-GB-1 and the TCTP-negative cell lines EH-GB-2 and SGC-996 (Fig. 2a). Treating NOZ, GBC-SD and OCUG-1 cells with DHA dramatically reduced migration (Fig. 3a and b). In contrast, DHA failed to inhibit migration in EH-GB-2 and SGC-996 cells (Fig. 3a and b). These findings indicate that DHA inhibits tumor cell migration in a TCTP-dependent manner. While these findings suggest an association between DHA’s anti-metastatic effects and TCTP status, tumor cell lines contain multiple mutations, each of which may also contribute to differential sensitivity to DHA. We engineered SGC-996 cells to over-express TCTP (Fig. 3c) and then treated these cells with DHA. SGC-996 cells that had acquired the expression of TCTP displayed 1.7-fold more cell migration than was observed in the cells expressing the control vector (Fig. 3d). However, in these cells, TCTP-dependent migration was completely blocked by treatment with DHA (Fig. 3d). In addition, while TCTP gene silencing reduced TCTP expression in NOZ cells (Fig. 3e), the cells in which TCTP was knocked down were not responsive to DHA and displayed no alterations in cell migration. In contrast, treating the control cells with DHA resulted in a 60% reduction in cell migration (Fig. 3f). Likewise, DHA abrogated invasion in TCTP-positive GBC-SD cells but did not block invasion in TCTP-negative EH-GB-1 cells (Additional file 1: Figure S1D). These data provide strong evidence supporting the hypothesis that DHA inhibits TCTP-mediated migration and invasion in GBC cells.

### DHA affect cell attachment, spreading, and focal adhesion

**Fig. 4:**
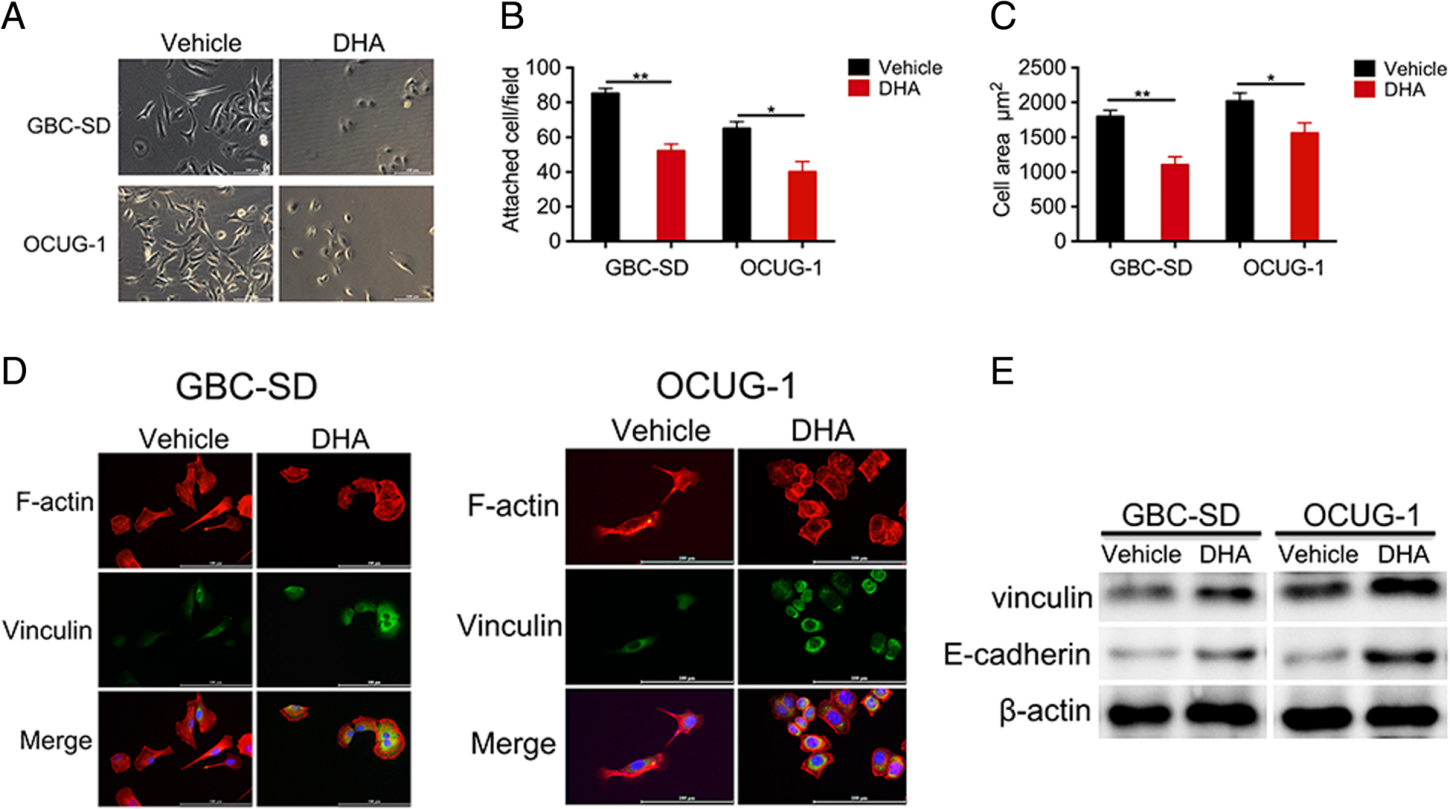
DHA altered cell attachment, spreading, and focal adhesions. **a** The morphologies of GBC-SD and OCUG-1 cells that were treated with DHA were examined under a microscope. **b** GBC-SD and OCUG-1 cells were treated with DHA for 2 days. They were then re-plated on fibronectin-coated coverslips for 6 h before being washed, fixed, and stained with rhodamine-conjugated phalloidin. The attached cells were counted using light microscopy. **c** GBC-SD and OCUG-1 cells were processed and stained as described in (**b**). Images of the cells were taken using a confocal microscope, and cell sizes were measured using Volocity software. At least 50 cells were measured for each time group. **d** Representative images of focal adhesions in GBC-SD and OCUG-1 cells that were treated with DHA. Cells were re-plated on fibronectin-coated coverslips, fixed, and stained with anti-vinculin antibodies and rhodamine-conjugated phalloidin. The cells were visualized using a confocal microscope. **e** Total cellular protein levels were detected using western blot analysis with antibodies against vinculin and E-cadherin. β-actin was used as an internal control

It is well known that tumor metastasis is a multi-step cell process that involves cell attachment, spreading and adhesion and is not limited to migration and invasion. We used in vitro assays to estimate the effect of DHA on specific parameters associated with cell metastasis. As shown in Fig. 4a, GBC cells treated with DHA became oval-shaped and displayed inhibited cell pseudopodia formation. Moreover, treating cells with DHA resulted in an approximately 1.6-fold lower rate of attachment to a fibronectin-coated surface in both GBC-SD and OCUG-1 cells than was observed in untreated cells (Fig. 4b). Consistent with the results of cell attachment experiments, treatment with DHA also resulted in a marked reduction in spreading in both cell types (Fig. 4c). In addition, significant changes were observed in cell morphology following treatment with DHA. Whereas control cells exhibited an elongated, fibroblast-like morphology, DHA-treated cells had a flattened cell phenotype (Fig. 4d). Focal adhesions are composed of dynamic supramolecular structures that connect the actin cytoskeleton with the extracellular matrix. We examined focal adhesions by staining cells with antibodies against vinculin and E-cadherin, which are markers of focal adhesions. DHA induced focal adhesions in GBC-SD and OCUG-1 cells and increased the expression levels of vinculin and paxillin (Fig. 4d and e).

### DHA reduces Cdc42 activation in TCTP-expressing GBC cells

**Fig. 5:**
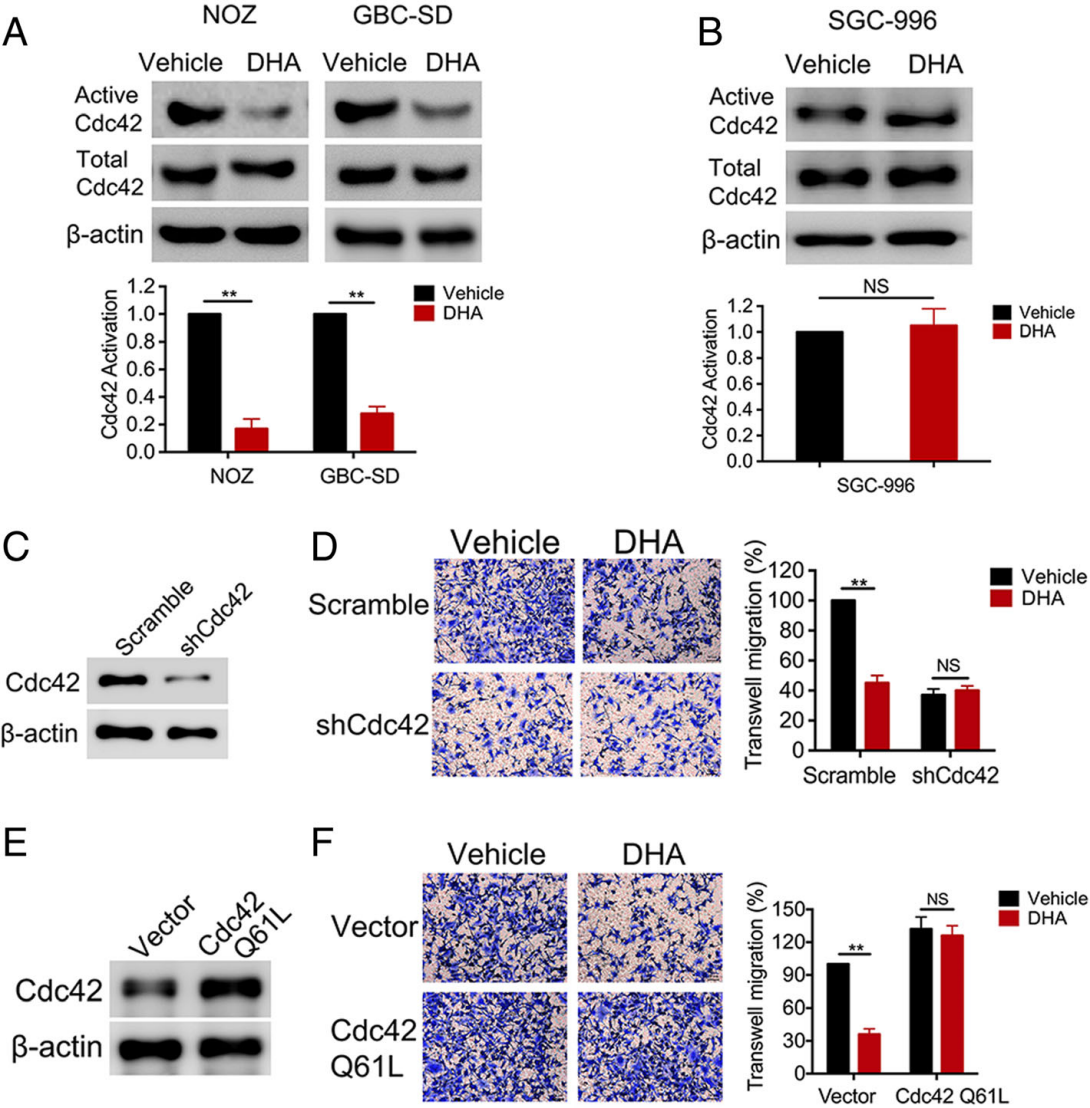
DHA reduces Cdc42 activation in TCTP-expressing cells. **a**, **b** NOZ and GBC-SD cells, which express TCTP, and SGC-996 cells, which do not express TCTP, were treated with DHA for 2 days. Cdc42 activation was analyzed using a configuration-specific monoclonal antibody that specifically recognizes Cdc42-GTP. The level of active Cdc42 was then quantified and normalized to the level of total Cdc42 in each sample, and all results were normalized to the level in the vehicle-treated control cells in each experiment. **c**, **d** NOZ cells were transfected with a negative scramble or a siRNA targeting Cdc42. The effect of knockdown was verified using immunoblotting. The NOZ cells were then treated with DHA for 2 days before they were seeded in transwell plates for migration assays. The relative migration rates are shown in a bar graph. **e**, **f** NOZ cells were transfected with an empty or myc-tagged active Cdc42 Q61L vector. Cdc42 Q61L overexpression was verified using immunoblotting. The NOZ cells were then treated with DHA for 2 days and plated in transwell plates for migration assays. The relative migration rates are shown in bar graph. The graphed data represent the mean ± SD of 3 independent experiments. *, *p* < 0.05; **, *p* < 0.01, ns: no significant difference (*p* > 0.05)

Our data showed that DHA inhibits GBC cell migration in a TCTP-dependent manner, and recent studies have demonstrated that TCTP activates Cdc42 [16]. We therefore sought to determine whether DHA inhibits Cdc42 activity in GBC cells. GBC cells were treated with DHA for 48 h, and Cdc42 activation was then analyzed using immunoblotting against Cdc42-GTP. Treatment with DHA resulted in an approximately 60–70% reduction in Cdc42 activation in NOZ and GBC-SD cells (Fig. 5a). However, DHA did not reduce Cdc42 activation in SGC-996 cells, which do not express TCTP (Fig. 5b). These data suggest that DHA inhibits the TCTP-mediated activation of Cdc42. We next performed experiments to explore whether Cdc42 is required for TCTP-mediated migration. First, Cdc42 was depleted in NOZ cells using gene knockdown (Fig. 5c). Similar to treatment with DHA (Fig. 3), knocking down Cdc42 reduced cell migration (Fig. 5d). However, DHA had no further inhibitory effect on Cdc42-depleted cells, suggesting that Cdc42 is required for DHA-mediated inhibitory effects. We next tested whether the inhibitory effects of DHA could be overcome by constitutive activation of Cdc42. NOZ cells were transfected with an empty vector or active Cdc42Q61L (Fig. 5e) and then treated with DHA for 48 h. They were then seeded in a migration transwell chamber. DHA-inhibited cell migration was completely reversed by the expression of active Cdc42Q61L (Fig. 5f). Taken together, these data suggest that in GBC cells, DHA inhibits cell migration by inactivating Cdc42.

### DHA reduces TCTP-dependent metastasis in vivo

**Fig. 6:**
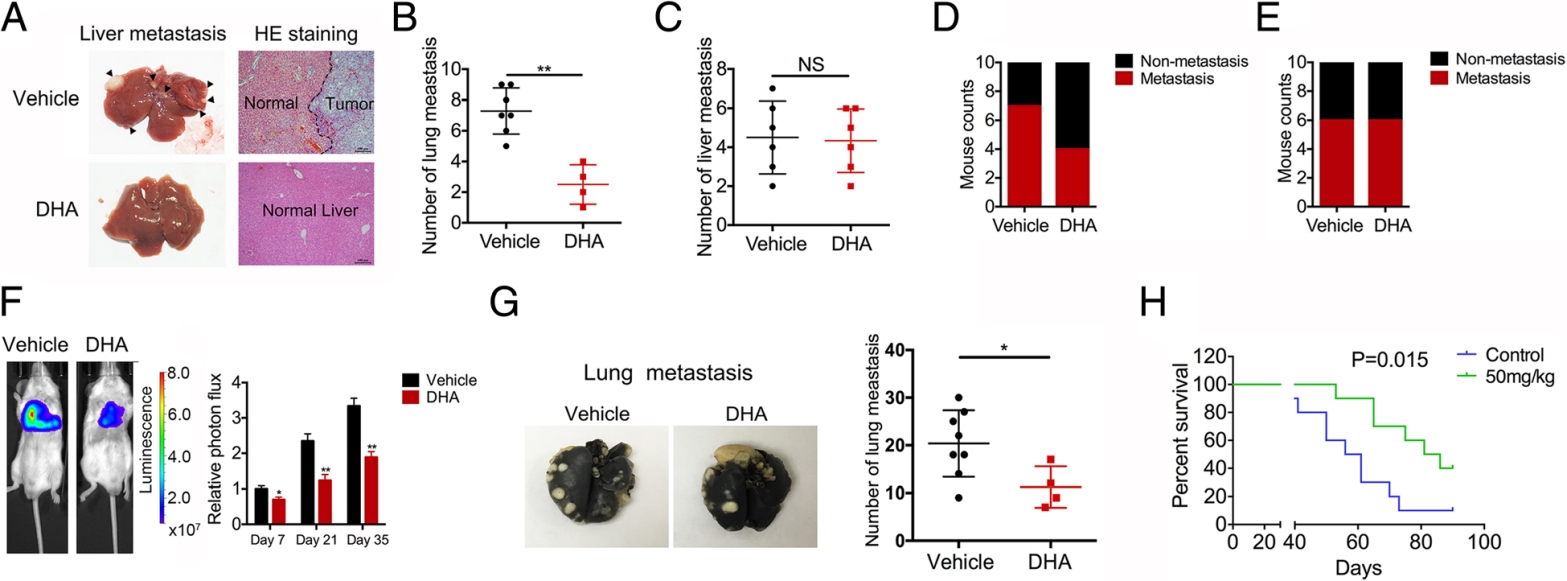
DHA reduces TCTP-dependent metastasis in vivo*.* **a** GBC cells were injected into the spleens of immunodeficient mice to establish a spleen-to-liver metastasis model, and the mice were then treated with DHA or vehicle control (PBS) via an IP injection every day. Representative photos of histological H&E-stained liver metastasis tissues are shown for each group. **b-c** A bar graph summarizing the number of liver metastases in the DHA-treated and control NOZ (**b**) and EH-GB-2 (**c**) cells. **d-e** A bar graph summarizing the incidence of liver metastasis in the DHA-treated and control NOZ (**d**) and EH-GB-2 (**e**) cells. **f** To establish a lung metastasis model, mice were intravenously injected into the tail with NOZ cells that expressed luciferase and then treated with DHA or vehicle control (PBS) via an IP injection every day. The bioluminescence of the cells was monitored every 2 weeks. Proton flux was evaluated using Xenogen IVIS LuminaXR software. The data shown represent the mean ± SD. **p < 0.001. NS: no significant difference. **g** Representative photos of histological lung metastasis tissues are shown for each group. A bar graph is used to summarize the number of lung metastases in the DHA-treated and control groups. **h** Kaplan–Meier plots of survival in the mice in the DHA-treated and control groups. Each group contained 10 mice

These earlier in vitro promising results suggest that DHA serves as an effective inhibitor to block TCTP-dependent GBC metastasis. To test this hypothesis, we established in vivo models of GBC metastasis. As liver metastasis accounts for the primary metastasis in GBC, liver metastasis models were induced using TCTP-positive NOZ and TCTP-negative EH-GB-2 cell lines. The GBC cells were injected into the spleen to establish a spleen-to-liver metastasis model in immunodeficient mice, which were subsequently treated with DHA or vehicle control (PBS) via an IP injection every day for 8 weeks. After the mice were euthanized, their livers were collected, and the number of macroscopic metastatic lesions was quantified. Metastases could be easily detected in the liver in the vehicle-treated control group (Fig. 6a). Significantly fewer metastases (50% fewer) were observed in the DHA-treated mice than in the vehicle-treated controls (Fig. 6b). Similarly, 4 out of 10 mice developed liver metastases in response to treatment with DHA, whereas 7 of 10 vehicle control mice developed liver metastases (Fig. 6d). In contrast, DHA had no effect on metastases in mice injected with the TCTP-negative cell line EH-GB-2 (Fig. 6c and e). To further evaluate the effect of DHA on GBC cell-distant metastases, we employed an animal model of experimental pulmonary metastasis. First, GBC cells were engineered to express firefly luciferase by infecting the cells with a lentivirus containing the corresponding recombinant plasmid. We then inoculated immunodeficient mice with the GBC cells via tail intravenous injections. The mice were subsequently treated with DHA or vehicle control (PBS) via IP injection every day. The resulting fluorescent signals were monitored every week. As shown in Fig. 6f, an increasing amount of proton flux was observed in the vehicle-treated mice, indicating that more tumor cells had migrated to the lung in this group than in the DHA-treated group. Upon necropsy, we found that treatment with DHA resulted in significantly fewer metastases than were observed in the vehicle-treated control mice (Fig. 6g). Consistent with this reduction in metastases, treatment with DHA increased survival times to 3–4 weeks long than was observed in the control mice (Fig. 6h). However, the DHA efficacy was not observed in tumor metastases developed from the TCTP-negative line EH-GB-2 (Additional file 1: Figure S1E-G). Taken together, these data support the hypothesis that DHA inhibits TCTP-mediated GBC metastases and improves mouse survival.

## Discussion

Patients with metastatic or relapsed GBC generally have a poor prognosis. Thus, significant improvement of new therapies is urgently needed for better outcomes of treatment of patients with GBC. TCTP expression drives multiple steps in tumorigenic and metastatic processes, suggesting that it could be a key node for therapeutic interventions [10, 16, 23]. Here, we show that DHA inhibits TCTP-dependent GBC cell metastasis. These data support the notion that a clinically viable DHA-based drug could be used as a novel anti-metastatic therapeutic agent in GBC patients.

DHA has been successfully used to treat malaria in a clinical setting for decades. In addition to its use as an effective anti-malarial drug, a growing number of studies have indicated that DHA also exerts anti-cancer effects in a variety of cancers [24]. DHA suppresses proliferation and angiogenesis and promotes apoptosis in pancreatic cancer cells via a microRNA-mRNA regulatory network [25]. DHA was demonstrated to have the ability to enhance the anti-pancreatic cancer effects of gemcitabine by inactivating NF-κB both in vitro and in vivo [26]. In addition, DHA has been shown to reduce both the incidence and the risk of metastasis in breast cancer and osteosarcoma [27, 28]. Moreover, DHA impedes metastasis in non-small-cell lung cancer by inhibiting the NF-κB/GLUT1 axis [29]. Here, we demonstrate that treating GBC cells with DHA both in vitro and in GBC-xenografted animal models dramatically reduced TCTP-dependent tumor metastasis. In addition, we found that DHA inhibited Cdc42-dependent tumor cell migration. Cdc42 is a small GTPase in the Rho subfamily that plays pivotal roles in cell morphology, migration, endocytosis, and cell-cycle progression. Additionally, the cytoskeletal rearrangements that are mediated by Cdc42 are important for metastasis [30–32], and activated Cdc42 phosphorylates the p21-activated kinases PAK1 and PAK2, which in turn initiate actin reorganization and regulate cell adhesion, migration, and invasion [33]. Interestingly, TCTP is also involved in regulating cell morphology through active interactions with the cytoskeletal protein actin [34], indicating that there is an intimate relationship between TCTP and Cdc42. All of these results indicate that a new therapeutic strategy could be centered on the ability of DHA to act as an anti-cancer agent that blocks cancer metastasis.

As a new class of anticancer drugs, DHA have many advantages, including well toleration, low toxicity to normal tissue/cells [35], low cross-drug resistance [36], and synergistic effects with many other traditional anti-cancer drugs [37]. Orally administered DHA is rapidly absorbed in the gastrointestinal tract, with Cmax achieved at approximately 1–2 h after administration [38]. Thus, DHA as an anti-metastatic therapeutic drug, may be used in combination with a means of targeting the primary tumor, such as resection or chemotherapy and/or radiation. Since some current methods of cancer treatment such as the inflammation following surgical resection or specific chemotherapeutic agents, are associated with an increase in metastatic dissemination [39, 40].

Although DHA inhibits cell migration and invasion during tumor metastasis, it is highly recommended to treat the disease at early stages. For instance, treatment of patients with DHA before the establishment of detectable metastases may minimize tumor further spreading or rapid recurrence. In our animal models, we found that DHA was effective against TCTP-positive but not against TCTP-negative tumors, suggesting that patients with TCTP-positive GBC may have great benefits from DHA.

However, even patients with seemingly TCTP-negative primary tumors may develop metastases from a subset of TCTP-positive cells. Clearly, a more complete understanding of the mechanisms underlying gallbladder cancer metastasis will be required to better define approaches to inhibit tumor dissemination.

## Additional file

Additional file 1: Figure S1. (A) TCTP expression was evaluated using IHC staining in 73 gallbladder cancer samples obtained from patients. A bar graph summarizes the number of TCTP-positive and -negative tissue samples. (B) Chemical structure of DHA. (C) DHA reduces TCTP expression levels in gallbladder cancer cells. Western blot analysis of TCTP proteins levels in the cell lysates of NOZ cells at 48 h after exposure to DHA (40 μM). β-actin was used as the loading control. (D) TCTP-positive GBC-SD and TCTP-negative EH-GB-2 cells were pre-treated with vehicle or DHA (40 μM) for 2 days and then seeded in transwells for 24 h for the invasion assays. (B) The relative invasion rates are shown in a bar graph. (E) The mice were intravenously injected with TCTP-negative EH-GB-2 cells expressing luciferase to establish a lung metastasis model and then treated with DHA or a vehicle control (PBS) via IP injections every day. The bioluminescence of the cells was monitored every 2 weeks. Proton flux was evaluated using Xenogen IVIS LuminaXR software. The data represent the mean ± SD. ***p* < 0.001. NS: no significant difference. (F) Representative photos of histological lung metastasis tissues are shown for each group. A bar graph summarizes the number of lung metastases in the DHA-treated and control groups. (G) Kaplan–Meier plots of survival in the mice in the DHA- and control-treated groups. (PNG 696 kb)
