# Correction to: Dihydroartemisinin inhibits TCTP-dependent metastasis in gallbladder cancer

**DOI:** 10.1186/s13046-019-1378-6

**Published:** 2019-09-03

**Authors:** Fei Zhang, Qiang Ma, Zihang Xu, Haibin Liang, Huaifeng Li, Yuanyuan Ye, Shanshan Xiang, Yijian Zhang, Lin Jiang, Yunping Hu, Zheng Wang, Xuefeng Wang, Yong Zhang, Wei Gong, Yingbin Liu

**Affiliations:** 1 Department of General Surgery, Xinhua Hospital affiliated to Shanghai Jiao Tong University School of Medicine, Room 517, Building 22, Xinhua Hospital, 1665 Kongjiang Rd, Shanghai, 200092 China; 2 Shanghai Research Center of Biliary Tract Disease, 1665 Kongjiang Road, Shanghai, 200092 China; 3 Laboratory of Integrative Medicine, School of Basic Medical Sciences, Shanghai University of Traditional Chinese Medicine, 1200 Cailun Road, Shanghai, 201203 China

**Correction to: J Exp Clin Cancer Res**

**http://dx.doi.org/10.1186/s13046-017-0531-3**

In the original publication of this article [1], there are mistakes in Fig. 3a and Fig. 3d.

**Fig. 3:**
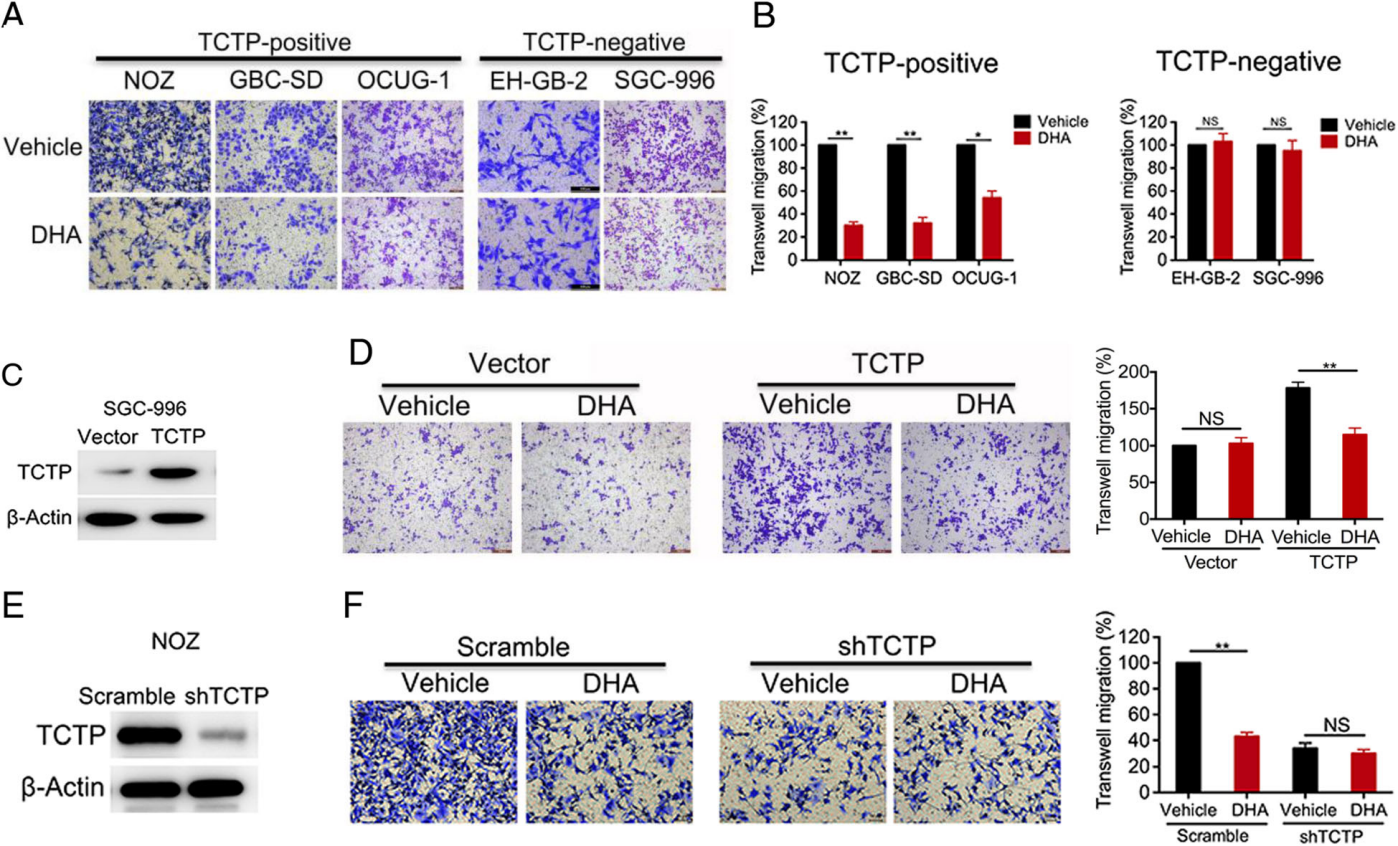
DHA inhibits TCTP-dependent cell migration and invasion. **a** In the migration assays, the TCTP-positive cell lines NOZ, GBC-SD, and OCUG-1, and the TCTP-negative cell lines EH-GB-2 and SGC-996 were pre-treated with either vehicle or DHA (40 μM) for 2 days and then seeded in transwell plates for 24 h. **b** The relative migration rates are shown in a bar graph. **c**, **d** SGC-996 cells were transfected with an empty or TCTP expression vector (**c**), treated with vehicle or DHA for 2 days, and then seeded in transwell plates for migration assays. The relative migration rates are shown in a bar graph (**d**). **e**, **f** TCTP was depleted in NOZ cells using shRNA (**e**). The cells were then treated with DHA for 2 days and seeded in transwell plates for migration assays. The relative migration rates are shown in a bar graph (**f**). The percentage of cells that migrated was scored and normalized to the percentage of migrated vehicle-treated cells. The graphed data represent the mean ± SD of 3 independent experiments. *, *p* < 0.05; **, *p* < 0.01, ns: no significant difference (*p* > 0.05)

The corrected Fig. 3a and Fig. 3d should be:
