# Correction to: Dihydroartemisinin inhibits TCTPdependent metastasis in gallbladder cancer

**DOI:** 10.1186/s13046-022-02325-1

**Published:** 2022-04-04

**Authors:** Fei Zhang, Qiang Ma, Zihang Xu, Haibin Liang, Huaifeng Li, Yuanyuan Ye, Shanshan Xiang, Yijian Zhang, Lin Jiang, Yunping Hu, Zheng Wang, Xuefeng Wang, Yong Zhang, Wei Gong, Yingbin Liu

**Affiliations:** 1 Department of General Surgery, Xinhua Hospital affiliated to Shanghai Jiao Tong University School of Medicine, Room 517, Building 22, Xinhua Hospital, 1665 Kongjiang Rd., Shanghai, 200092 China; 2 Shanghai Research Center of Biliary Tract Disease, 1665 Kongjiang Road, Shanghai, 200092 China; 3 Laboratory of Integrative Medicine, School of Basic Medical Sciences, Shanghai University of Traditional Chinese Medicine, 1200 Cailun Road, Shanghai, 201203 China

**Correction to: J Exp Clin Cancer Res 36, 68 (2017)**

**https://doi.org/10.1186/s13046-017-0531-3**

Following publication of the original article [1], the authors identified minor errors in Figs. 3, 6, and S1; specifically:

- Fig. 3A: Incorrect migration assay images originally used for the TCTP-positive cell lines GBC-SD; the correct images are now used, and a scale bar has been added
- Fig. 6G: scale bars have been added to the images showing lung metastatic tissues of gallbladder cancer perfused with Indian ink
- Fig. S1F: scale bars have been added to the images showing lung metastatic tissues of gallbladder cancer perfused with Indian ink

The corrected figures are given here. The correction does not have any effect on the final conclusions of the paper.

**Fig. 3:**
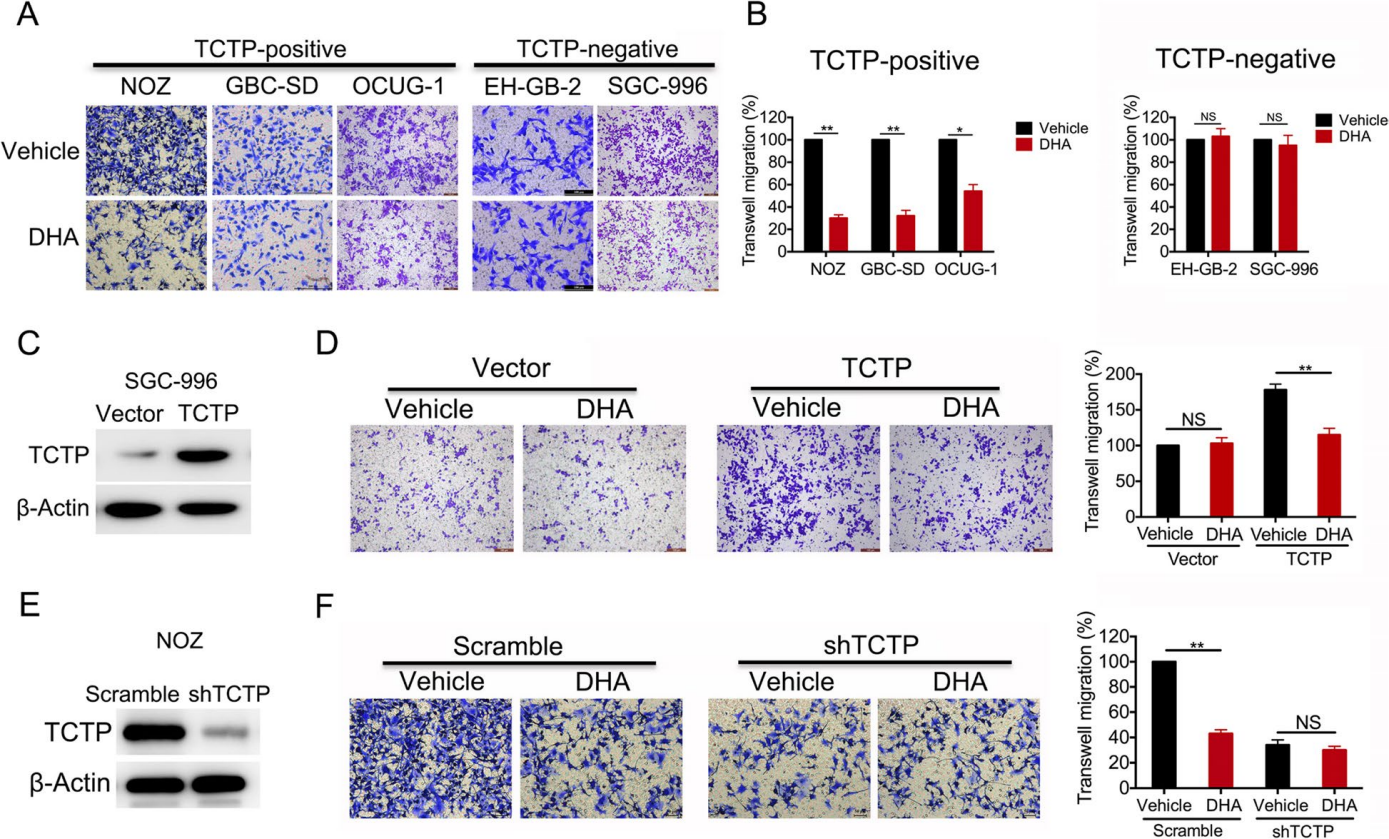
DHA inhibits TCTP-dependent cell migration and invasion. **a** In the migration assays, the TCTP-positive cell lines NOZ, GBC-SD, and OCUG-1, and the TCTP-negative cell lines EH-GB-2 and SGC-996 were pre-treated with either vehicle or DHA (40 μM) for 2 days and then seeded in transwell plates for 24 h. **b** The relative migration rates are shown in a bar graph. **c, d** SGC-996 cells were transfected with an empty or TCTP expression vector (**c**), treated with vehicle or DHA for 2 days, and then seeded in transwell plates for migration assays. The relative migration rates are shown in a bar graph (**d**). **e**, **f** TCTP was depleted in NOZ cells using shRNA (**e**). The cells were then treated with DHA for 2 days and seeded in transwell plates for migration assays. The relative migration rates are shown in a bar graph (**f**). The percentage of cells that migrated was scored and normalized to the percentage of migrated vehicle-treated cells. The graphed data represent the mean ± SD of 3 independent experiments. *, *p* < 0.05; **, *p* < 0.01, ns: no significant difference (*p* > 0.05)

**Fig. 6:**
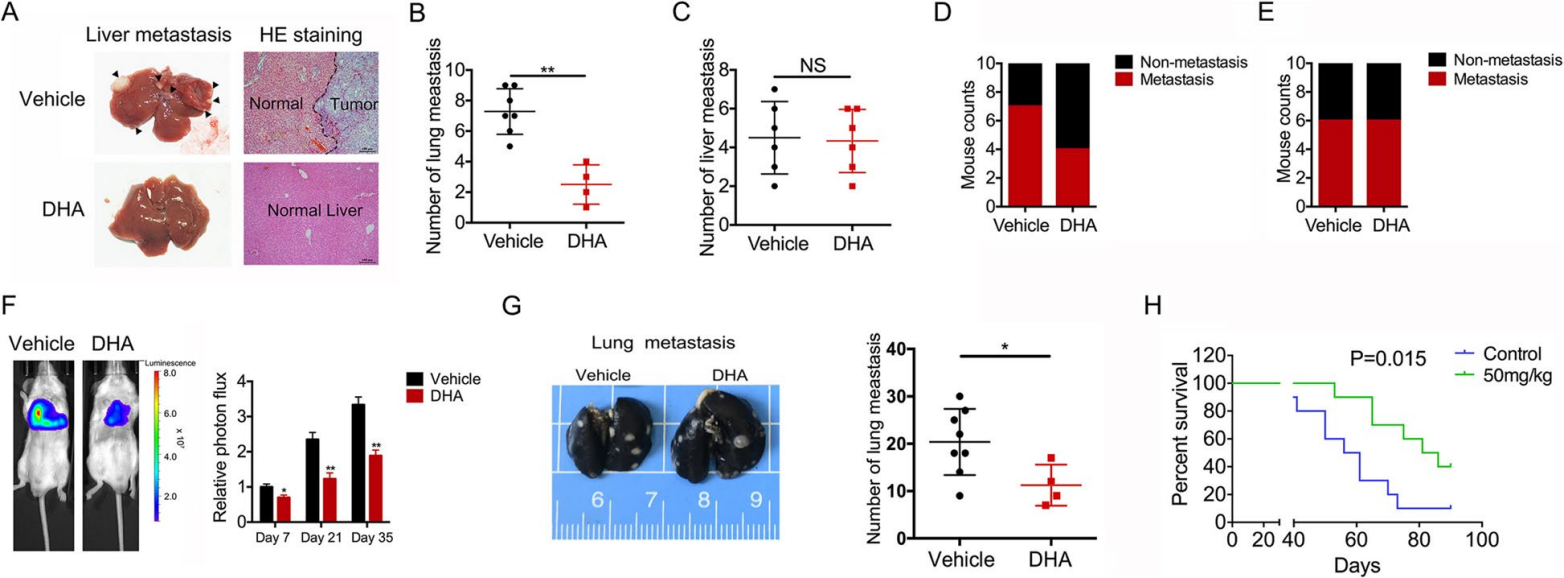
DHA reduces TCTP-dependent metastasis in vivo. **a** GBC cells were injected into the spleens of immunodeficient mice to establish a spleen-to-liver metastasis model, and the mice were then treated with DHA or vehicle control (PBS) via an IP injection every day. Representative photos of histological H&E-stained liver metastasis tissues are shown for each group. **b-c** A bar graph summarizing the number of liver metastases in the DHA-treated and control NOZ (**b**) and EH-GB-2 (**c**) cells. d-e A bar graph summarizing the incidence of liver metastasis in the DHA-treated and control NOZ (**d**) and EH-GB-2 (**e**) cells. **f** To establish a lung metastasis model, mice were intravenously injected into the tail with NOZ cells that expressed luciferase and then treated with DHA or vehicle control (PBS) via an IP injection every day. The bioluminescence of the cells was monitored every 2 weeks. Proton flux was evaluated using Xenogen IVIS LuminaXR software. The data shown represent the mean ± SD. **p < 0.001. NS: no significant difference. **g** Representative photos of histological lung metastasis tissues are shown for each group. A bar graph is used to summarize the number of lung metastases in the DHA-treated and control groups. **h** Kaplan–Meier plots of survival in the mice in the DHA-treated and control groups. Each group contained 10 mice

## Supplementary Information

**Additional file 1: Figure S1.** (A) TCTP expression was evaluated using IHC staining in 73 gallbladder cancer samples obtained from patients. A bar graph summarizes the number of TCTP-positive and -negative tissue samples. (B) Chemical structure of DHA. (C) DHA reduces TCTP expression levels in gallbladder cancer cells. Western blot analysis of TCTP proteins levels in the cell lysates of NOZ cells at 48 h after exposure to DHA (40 μM). β-actin was used as the loading control. (D) TCTP-positive GBC-SD and TCTP-negative EH-GB-2 cells were pre-treated with vehicle or DHA (40 μM) for 2 days and then seeded in transwells for 24 h for the invasion assays. (B) The relative invasion rates are shown in a bar graph. (E) The mice were intravenously injected with TCTP-negative EH-GB-2 cells expressing luciferase to establish a lung metastasis model and then treated with DHA or a vehicle control (PBS) via IP injections every day. The bioluminescence of the cells was monitored every 2 weeks. Proton flux was evaluated using Xenogen IVIS LuminaXR software. The data represent the mean ± SD. **p < 0.001. NS: no significant difference. (F) Representative photos of histological lung metastasis tissues are shown for each group. A bar graph summarizes the number of lung metastases in the DHA-treated and control groups. (G) Kaplan–Meier plots of survival in the mice in the DHA- and control-treated groups.
