# Supplementary figures and images for: Dihydroartemisinin inhibits TCTP-dependent metastasis in gallbladder cancer
Source: J Exp Clin Cancer Res. 2017 May 15;36:68. doi: 10.1186/s13046-017-0531-3 (PMC5433060; doi:10.1186/s13046-017-0531-3)

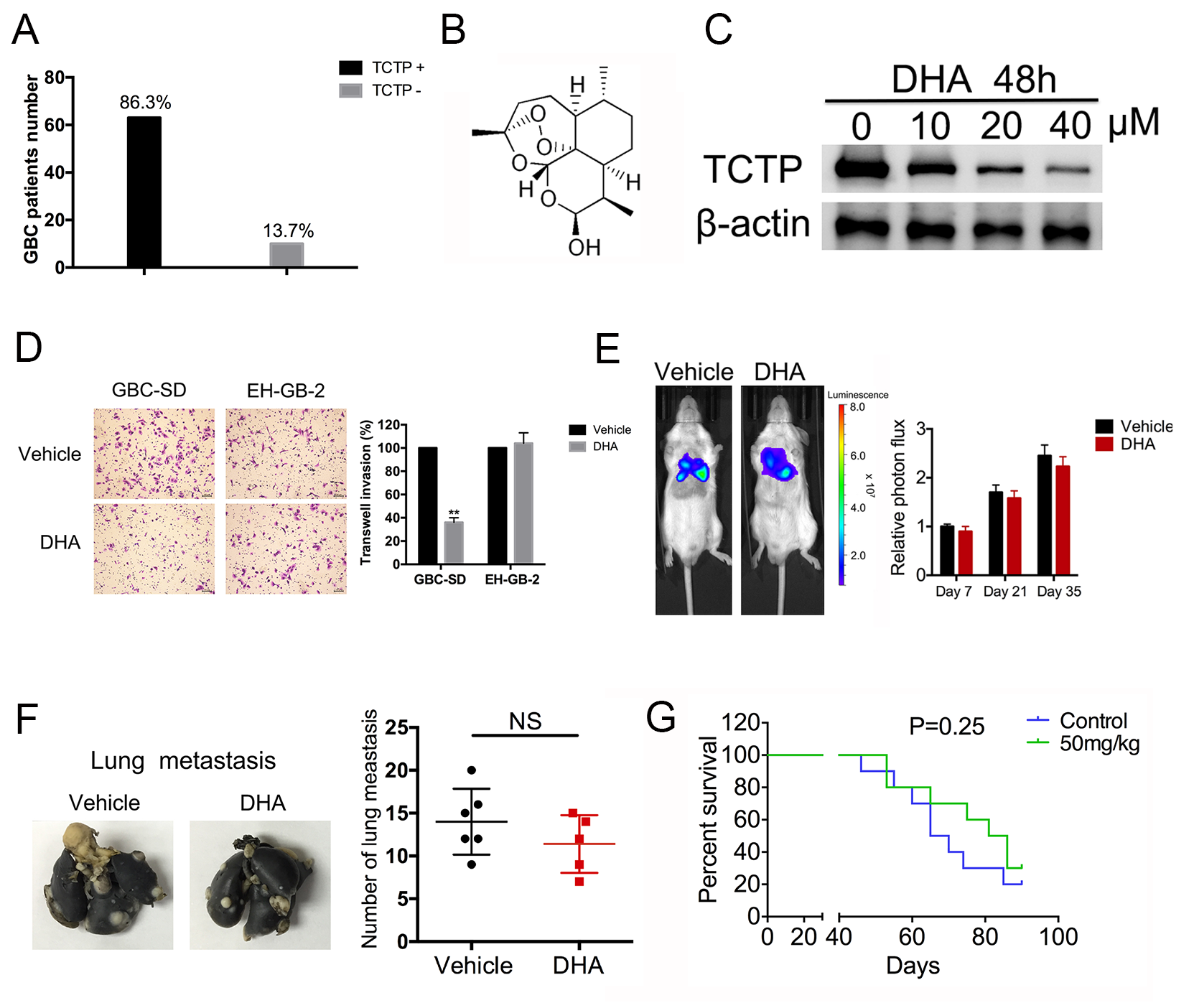

Supplement: Additional file 1: Figure S1. — (A) TCTP expression was evaluated using IHC staining in 73 gallbladder cancer samples obtained from patients. A bar graph summarizes the number of TCTP-positive and -negative tissue samples. (B) Chemical structure of DHA. (C) DHA reduces TCTP expression levels in gallbladder cancer cells. Western blot analysis of TCTP proteins levels in the cell lysates of NOZ cells at 48 h after exposure to DHA (40 μM). β-actin was used as the loading control. (D) TCTP-positive GBC-SD and TCTP-negative EH-GB-2 cells were pre-treated with vehicle or DHA (40 μM) for 2 days and then seeded in transwells for 24 h for the invasion assays. (B) The relative invasion rates are shown in a bar graph. (E) The mice were intravenously injected with TCTP-negative EH-GB-2 cells expressing luciferase to establish a lung metastasis model and then treated with DHA or a vehicle control (PBS) via IP injections every day. The bioluminescence of the cells was monitored every 2 weeks. Proton flux was evaluated using Xenogen IVIS LuminaXR software. The data represent the mean ± SD. **p < 0.001. NS: no significant difference. (F) Representative photos of histological lung metastasis tissues are shown for each group. A bar graph summarizes the number of lung metastases in the DHA-treated and control groups. (G) Kaplan–Meier plots of survival in the mice in the DHA- and control-treated groups. (PNG 696 kb) [file 13046_2017_531_MOESM1_ESM.png]
